# 

**Published:** 1920-08-14

**Authors:** 


### Gifts and Bequests.

East Suffolk Hospital will receive £250 under the will of Mr. J. A. Burgess, of Melton, Suffolk.

Captain Nevill Reeve-King, of Ashby Hall, Lincoln, has left £100 to Lincoln County Hospital.

Mr. T. W. Backhouse, a prominent Quaker, of Sunderland, has bequeathed £1,000 to Sunderland Royal Infirmary.

Mr. Harrison Mudd, of Great Grimsby, has left £100 each to the Grimsby and District Hospital and the London Temperance Hospital.

Mr. J. A. Mills, of South Lowestoft, left the reversion of his property, valued at nearly £50,000, to the Norfolk and Norwich Hospital, St. Dunstan's Hostel, and three other institutions.

Under the will of the late Mr. J. T. G. Clarke, J.P., of Leicester, the Royal Infirmary has been left £1,000, the Hospital Saturday Society £500, and the Surgical Aid Society £200.

Dr. Diggle, the late Bishop of Carlisle, has directed that after the death of his wife £1,000 shall be paid to Cumberland Infirmary, Carlisle, to endow a bed or beds for the special benefit of sufferers from rodent ulcer or cancer.

Mr. William Ward, of Blackburn, has left £10,000 for such local charitable objects as his executors shall select, and after a number of other bequests have been paid the residue of his estate to the Blackburn Royal Infirmary.

Under the will of Mr. Lewis Atkinson, of Higher Broughton, Manchester, £2,000 has been left to the Manchester Royal Infirmary, £1,200 to the Salford Royal Hospital, £1,000 to St. Mary's Hospital Manchester and £500 to the Manchester Eye Hospital. The residue of the estate is left to such charitable objects as the trustees shall select.

### The South-Eastern Hospital for Children.

Her Royal Highness Princess Beatrice has graciously intimated that she will become a patroness of the above institution. This hospital, which has throughout its history shown healthy progress, was opened as a convalescent home in 1872 for the reception of six children recovering from operations or illnesses. It has now grown to be a children's hospital of sixty beds. It receives over 500 in-patients and treats over 10,000 out-patients annually.

### Matrons' and Sisters' Appointments.

Bulwell Hall Sanatoria for Children, Nottingham.—Miss M. E. Dale has been appointed matron. She was trained at Victoria Hospital, Richmond, Yorkshire, has been staff nurse at the Royal Hospital for Sick Children, Edinburgh, sister at Oldham Royal Infirmary, night sister at the Royal Albert Edward Infirmary, Wigan, assistant matron of the Orthopædic Hospital, Baschurch, Shrewsbury, and has served abroad under the Queen Alexandra's Imperial Military Nursing Service Reserve.

District Hospital, Minehead.—Miss Ethel Dowson has been appointed matron. She was trained at the Royal Infirmary, Bradford, and has since been sister at St. Bartholomew's Hospital, Rochester, and home sister and assistant matron at Warneford and Leamington General Hospital.

Dorset Mental Hospital.—Miss E. M. Yeadon has been appointed night sister. She was trained at the Royal Portsmouth Hospital and at the Graylingwell Mental Hospital, Chichester, has been ward sister at the Kent and Canterbury Hospital, ward and night sister at the County Hospital, Lincoln, night sister and housekeeping sister at the Royal Portsmouth Hospital, and has also done private nursing. Miss Yeadon holds the Medico-Psychological certificate and is a member of the College of Nursing.

Princess Mary's Hospital for Children, Margate.—The Misses F. B. Roberts, V. V. Blake, and G. M. McClemens have been appointed staff nurses. The Misses Roberts and Blake were trained at the Royal Devon and Exeter Hospital, Exeter, and Miss McClemens at Guy's Hospital, where she has since been staff nurse. She holds the certificate of the Central Midwives Board.

Queen Mary's Convalescent Auxiliary Hospital for Limbless Men, Roehampton.—Miss Jessie Monroe Kirkpatrick has been appointed assistant matron. She was trained at the David Lewis Northern Hospital, Liverpool, has been housekeeping sister at the General Hospital, Swansea, night sister at the West Kent General Hospital, Maidstone, and is a member of Queen Alexandra's Imperial Military Nursing Service Reserve.

Victoria Cottage Hospital, Romford.—Miss Annie G. Duxfield has been appointed matron. She was trained at Sheffield Union Hospital, and has since been staff nurse at Jessop Hospital, Sheffield, ward sister at Fulham Infirmary, sister-in-charge, theatre sister, and assistant matron at Ilford Emergency Hospital. She holds the certificate of the Central Midwives Board.

Miss Dorothy de M. Rudolf, who has had wide experience as Secretary of the Children's Union of the Waifs and Strays Society for the past five years, has been appointed Appeal Secretary, and is to take up her duties at the hospital early in September.

### New Buildings Contemplated.

Leamington.—A portion of the money raised as a War Memorial will be devoted to the enlargement of the Warneford Hospital, Leamington. An enlargement scheme at the County Hospital, costing £10,300, has been approved. It includes the completion and equipment of the nurses' house, Merthyr. Mr. E. M. G. Richards and Mr. C. M. Davies have been appointed architects to prepare plans, in conjunction with the medical staff, for further hospital accommodation. Mr. Seymour Barry has offered to spend £10,000 on an immediate temporary building, and to give £20,000 towards a new general hospital, provided that a maintenance fund of £100,000 is raised.

Acton.—A scheme costing £12,000 has been adopted for the extension of the hospital. The honorary architect is Mr. H. C. H. Monson Braintree. The Urban District Council has passed plans for the Cottage Hospital to be erected by Mr. W. J. Courtauld in London Road.

Fraserburgh.—A gift of £1,000 has been received for building an operating theatre in the Thomas Walker Hospital.

Penrith.—Funds are being raised for extensions of the Cottage Hospital.

Peterborough.—It has been decided to throw open, under the rules and regulations of the Royal Institute of British Architects, the designing of the proposed War Memorial Hospital for Peterborough. The building is estimated to cost £75,000, and the total expenditure will probably not be less than £100,000.

Keighley.—Taking down, removal, and reconstruction of hospital buildings for the Keighley and Bingley Joint Hospital Board has been entrusted to the architects, Messrs. Moore and Crabtree.

Durham.—Plans are being prepared by Mr. E. T. Hall, of London, for extensions to the Durham County Hospital. It is stated that the work will involve a cost of £130,000.

Hitchin.—An addition of sixteen beds and other improvements are being considered.

Horsham.—The annual meeting of the Horsham Cottage Hospital authorised the preparation of plans for a building to accommodate thirty beds.

Worthing.—The Hospital Committee proposes to erect a maternity wing at an estimated cost of £6,000.

Chapel-en-le-Frith.—A scheme is afoot for the provision of a cottage hospital for the Chapel-en-le-Frith and Chinley districts.

The North Staffordshire Cripples' Aid Society, at a recent meeting, decided to commence their rebuilding and reconstruction scheme, and approved plans for the construction of additional nurses' accommodation. The contract is for £3,850.

### The Book Market.

Angus and Robertson, Ltd., Sydney, and Oxford University Press: "Letters of an Australian Army Sister." By Anne Donnell. 6s. net.

H. Frowde and Hodder and Stoughton: "Manual of Medicine." By A. S. Woodwark, C.M.G., C.B.E., M.D., F.R.C.P. Second edition. 16s. net.

Macmillan and Co., Ltd.: "Notes on a Cellar-Book." By George Saintsbury. 7s. 6d. net.

John Murray: "The Shibboleths of Tuberculosis." By M. Paterson, M.D. 10s. 6d. net.

Henry Frowde and Hodder and Stoughton: "Infectious Diseases." By C. B. Ker, M.D., F.R.C.P. £2 2s. net.

John Bale, Sons and Danielsson, Ltd.: "Gonococcal Infection in the Male." By Norman Lumb, O.B.E., M.B., M.R.C.S., L.R.C.P. 25s. net. "Military Sanitation." By Col. R. J. Blackham, C.B., C.M.G., C.I.E., D.S.O., etc. Third edition. 10s. 6d. net.

